# An Essay on the Treatment and Cure of Pulmonary Consumption, on Principles Natural, Rational, and Successful

**Published:** 1840-10

**Authors:** 


Art. IV.—*An Essay on the Treatment and Cure of Pulmonary Consumption, on principles natural, rational, and successful: with suggestions for an improved plan of treatment of the disease among the lower classes of society; and a relation of several successive cases restored from the last stage of consumption to a good state of health.* By George Boddington, Surgeon.—*London*, 1840. 12mo, pp. 60.

If this lengthy and ingenious title-page is calculated to excite painful suspicions in the mind of the experienced reader, as to the nature of the work and the object of the author, the perusal of the book itself most assuredly will not allay them. It is, in fact, an elaborate advertisement to make known to all concerned, that the author has "taken a large house near his own residence, for the reception of patients," &c. &c. (Introd. p. viii.) To this house and to its renter's care the author is desirous that the medical gentlemen of large towns "should confide their consumptive patients, instead of sending them, as many now do, to take their chance, or probably to fall into the hands of *mercenaries* [!] at some distant seaport where they commonly die, far away from friends and home." (p. 45.) We will only say further of this book, that it is consolatory to know, after knowing its object, that it betrays an utter ignorance of pathology, of therapeutics, and of the English language. We will not give the name of Mr. Boddington's abode, for fear we should be charged advertisement duty for this notice of his book.

